# Applying a Green Solvent with Microwave, Ultrasound, and Soxhlet Extraction Techniques to Quantify the Slip Additive *cis*-1,3-Docosenamide and Nine Oxidative Degradation Byproducts in Polypropylene Samples

**DOI:** 10.3390/polym15163457

**Published:** 2023-08-18

**Authors:** Hernández Fernández Joaquin, Pérez Mendoza Jaime, Ortega-Toro Rodrigo

**Affiliations:** 1 Chemistry Program, Department of Natural and Exact Sciences, San Pablo Campus, University of Cartagena, Cartagena 130015, Colombia; 2 Chemical Engineering Program, School of Engineering, Universidad Tecnológica de Bolivar, Parque Industrial y Tecnológico Carlos Vélez Pombo Km 1 Vía Turbaco, Cartagena 130001, Colombia; 3 Department of Natural and Exact Science, Universidad de la Costa, Barranquilla 080002, Colombia; 4 Complex Fluid Engineering and Food Rheology Research Group (IFCRA), Food Engineering Department, Universidad de Cartagena, Cartagena de Indias 130015, Colombia; jperezm@unicartagena.edu.co; 5 Food Packaging and Shelf-Life Research Group (FP&SL), Food Engineering Department, Universidad de Cartagena, Cartagena de Indias 130015, Colombia; rortegap1@unicartagena.edu.co

**Keywords:** erucamide, extraction, microwave, Soxhlet, ultrasound, cyclohexane, dichloromethane, limonene, GC-MS

## Abstract

Erucamide is used as an important slip agent for polymers. However, erucamide can degrade during processing and long-term storage, forming various oxidation products. These degradation products can affect the recovery rates of erucamide. In this study, investigated different solid–liquid extraction methods (Soxhlet, microwave, and ultrasound) and used gas chromatography with mass spectrometry (GC-MS) to quantify erucamide and its degradation byproducts in polypropylene (PP). A multivariable experiment was designed, and a mixed-effect approach was used to analyze the results. Various extraction variables were examined, such as temperature, time, solvents, and PP pretreatments. Using a mixed-effect model with a Kenward–Roger approximation, an R2 of the model of 97% and *p* values of 0.168, 0.000, and 0.000 were obtained for the technical, solvent, and type of PP pretreatment variables, respectively. The highest average recoveries of erucamide were found with the microwave technique and were 96.4% using dichloromethane, 94.57% using cyclohexane, and 93.05% using limonene. With ultrasound, recoveries ranged between 85 and 92% for dichloromethane and limonene. In addition, it was observed that the extraction method had better recovery results in ground PP than in films and in pellets. Nine oxidative degradation byproducts of erucamide were identified and semi-quantified by GC-MS. The reaction mechanisms for forming each byproduct were proposed. The byproducts that experienced a higher rate of degradation of erucamide were erucamide with a hydroxyl group at position one and 12-amino-6-12-oxo-dodecanoic acid, showing more prominent peaks using the Soxhlet method with cyclohexane and dichloromethane as solvents and polypropylene (PP) films as the type of material used.

## 1. Introduction

There is a tremendous demand for polypropylene films with different characteristics, such as multilayer films, materials for controlled atmosphere, and barrier materials, especially in the packaging industry [1,2,3,4,5]. In their various formulations, these films must meet intrinsic and extrinsic requirements for their preparation, handling, and application and for their final use [6,7,8,9,10]. There are two fundamental functions that these films must fulfill. In the first place, they must be easily manipulated by the corresponding machinery in the packaging or packaging lines without the occurrence of phenomena such as electrification or adhesion between the film and the product, between films, or between the film and any part of the machine [11,12,13,14]. This implies that they must have good sliding and anti-blocking properties. To achieve this, during the industrial manufacture of the films and their incorporation into the raw materials, additives known as slip and anti-block agents are added. Among the commonly used slip additives in polypropylene (PP) are long-chain fatty acid amides [15,16,17,18]. These additives help to reduce friction and facilitate the sliding of the films during processing and handling, avoiding problems such as blocking or unwanted adhesions [19].

Erucamide is an amide belonging to the monounsaturated fatty acids group, characterized by a hydrocarbon chain composed of 22 carbon atoms in its chemical structure [15,20]. Erucamide is thermally stable at a relatively higher temperature than other slip agents, such as oleamide [15]. During the extrusion and molding of PP, erucamide dissolves in the polymer. Then erucamide crystallizes in the polymer to form a lubricating layer on the surface of the solidified polymer [15]. During the erucamide dosing process, erucamide may be added along with anti-blocking agents such as silica or talc; in such cases, erucamide is absorbed onto the surface of the anti-blocking agent [15]. As a combination, erucamide, and silica reduce the coefficient of friction of PP more effectively than either alone [1]. As a general rule, it is essential to note that the most effective slip agent is only sometimes the best anti-blocking agent. In this context, erucamide is a slip and anti-block additive that is preferred in PP-producing industries due to its higher melting point and higher heat resistance [20]. These properties make erucamide more suitable for applications where higher thermal stability and resistance to surface blocking are required than PP films [21,22,23,24].

Traditionally extraction of erucamide from polymers has been performed by Soxhlet extraction, ultrasonic-assisted extraction (UAE) [25], supercritical fluid extraction (SFE) [26], and microwave-assisted extraction (MAE) [26,27]. Although Soxhlet extraction achieves good efficiency, it is slow [27]. On the other hand, ultrasonic and microwave extraction are environmentally friendly techniques that offer several advantages over conventional methods. These advantages include reduced extraction time, lower solvent use, and higher yield of the chemical of interest. These ecological techniques allow more efficient and sustainable extractions. Some of these techniques have been used individually to extract erucamide. Still, these extraction techniques have not been studied simultaneously with green solvents, and the effect of each extraction technique on the oxidative degradation of erucamide during its extraction from the PP matrix has not yet been investigated [28,29,30,31,32].

**Figure 1:**
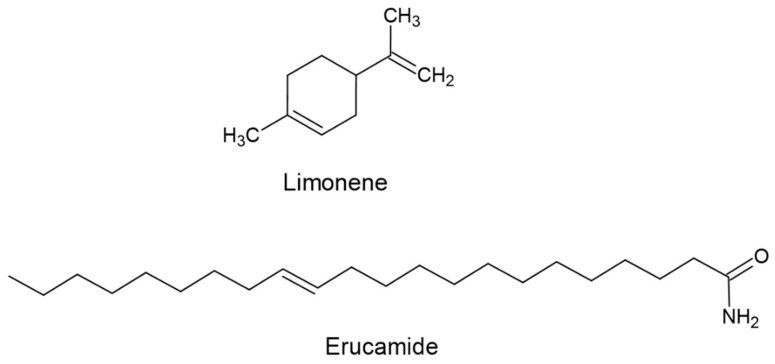
Chemical structure of erucamide and limonene.

Selection of the extraction solvent is crucial to achieving complete extraction, preventing it from reacting with erucamide, preventing it from completely solubilizing the polymer, determining the duration of extraction in each of the extraction techniques, and swelling the polymer. And improve the miscibility and diffusion within the polymer. This is important because as the polymer swells, the solvent penetrates the intermolecular spaces of the polymer, separating the polymer chains and allowing the erucamide to be extracted to dissolve more quickly in the solvent [33]. Commonly, solvents such as cyclohexane and dichloromethane are used in solid–liquid extraction [34,35]. However, cyclohexane has occupational and environmental disadvantages due to its rapid absorption in the human body [36]. On the other hand, dichloromethane has been reported to have harmful health and environmental effects, including the risk of diseases such as brain and liver cancer and DNA damage [37,38,39,40,41]. Due to the potential dangers associated with cyclohexane and dichloromethane, safer and more environmentally friendly alternatives are sought, such as green solvents [37,38,39,40,41,42,43,44]. One of the leading candidates as a green solvent is limonene, which belongs to the chemical family of monoterpenes (Figure 1) and is a natural compound.

Limonene is known as an ecological solvent and, therefore, an excellent substitute for hazardous solvents in multiple aspects [45,46,47,48]. Limonene is a colorless liquid that has a high capacity to dissolve both polar and nonpolar substances, making it ideal for use as a solvent in industrial applications. Limonene is biodegradable and non-toxic, making it safer and more environmentally friendly than many synthetic solvents. It is beneficial as a solvent in the cleaning industry and is used in household and commercial cleaning products. Limonene as a solvent has also been explored in other applications, including pharmaceuticals, food and beverages, and extracting organic compounds from plants. Limonene as a biosolvent is considered a sustainable and efficient alternative to traditional solvents, making it an increasingly popular option in the industry [49,50,51,52,53]. Based on the latest EU OEL data, no specific occupational exposure limit is set for limonene. However, the MAK value (Maximale Arbeitsplatzkonzentration) of 28 mg/m^3^ is reported, which refers to the occupational exposure limit established for limonene by the Hazardous Substances Committee of the German Research Foundation for Occupational Safety and Health (Deutsche Forschungsgemeinschaft (DFG)) [54]. This value can be considered relatively “good” compared to the values reported for cyclohexane and dichloromethane. It is important to note that MAK values are specific to Germany and may vary in other countries or regions. However, the MAK value of 28 mg/m^3^ can be a valuable reference to assess and monitor exposure to limonene in the workplace [54]. Limonene is an excellent biosolvent due to its chemical and physical properties, such as its dipole moment, electronegativity, and reactivity. These physicochemical properties should allow a very low reactivity and degradation of erucamide. This investigation evaluated the effect of dichloromethane, cyclohexane, and limonene on the degradation of erucamide.

In our research, we performed microwave-assisted solvent extraction, ultrasonic-assisted extraction, and Soxhlet extraction based on a sensitive microextraction method, flame ionization detector gas chromatography, and mass spectrometry. In each extraction technique, the erucamide recovery percentage was evaluated, and the extent to which each method affects the formation of erucamide oxidation or degradation byproducts was determined. For the degradation byproducts formed, we propose respective reaction mechanisms that allow us to understand how erucamide degradation occurs. We carried out three previous PP treatments (ground PP, PP granules, and PP films) using three different solvents (dichloromethane, cyclohexane, and limonene) and determined how each of these procedures affects the generation of nine additive degradation byproducts. The performance of the multiple variables was evaluated through multivariate statistical analysis, allowing the development of a multiple regression model that allows a comprehensive understanding and control of most factors present in the quantification of erucamide in polypropylene. This research should support other researchers, polypropylene-producing industries, film manufacturers, erucamide-producing industries, and chemical regulators interested in quantifying erucamide from a polymeric matrix and knowing its performance as a slip and anti-blocking agent.

## 2. Materials and Methods

### 2.1. Materials and Reagents

The working cis-1,3-docoseneamide (erucamide) was supplied by Cymit Quimica Croda Universal (4014 Walnut Pond Drive Houston, TX 77059 281-282-0022 Crompton (Witco) Corporation) (erucamide has a white coloration; its iodine value ranged from 75 to 82. The result of the acid number was 0.1 KOH mg/g, and the pour point ranged from 78 to 81 °C. The moisture content obtained was 0.4% max. The certified purity was 99% min). N-tetradecanamide (Alpha Aesar, Karlsruhe, Germany) was used as internal standard. Limonene (HPLC grade) was obtained from Scharlab (Barcelona, Spain). Hydrogen 99.9999% was from Linde (Cartagena, Colombia), nitrogen 99.9999% was from Linde (Cartagena, Colombia), cyclohexane 99.5% was from Panreac (Barcelona, Spain), and dichloromethane 99.99% was from Sigma Aldrich (Bangalore, India).

### 2.2. Instrumentation

An Agilent 6890 gas chromatograph (GC) (Agilent Technologies, Wilmington, CA, USA) with a mass detector (MS) was used to measure the samples. The MS detector was heated to 230 °C. An Agilent J&W VF-5 ms column (5% phenyl and 95% dimethylpolysiloxane) with 30 m × 0.25 mm i.d., with a diameter of 0.25 m, was used. The oven heating cycle started at 200 °C for 4 min, rose to 280 °C at a rate of 10 °C min^−1^, and remained there for 7 min. Helium, at 1.0 mL min^−1^, was the carrier gas (99.996%). The injection system was in splitless mode. One µL of the sample was injected. The GC-MS apparatus was operated and the data were processed with Chemstation software. With these chromatographic parameters, it was possible to obtain a retention time (T_r_) of 7.5 min for erucamide.

#### 2.2.1. Preparation of Erucamide Calibration Standards and PP Samples with Erucamide

##### Preparation of the Curve for Chromatograph Calibration

**Figure 2:**
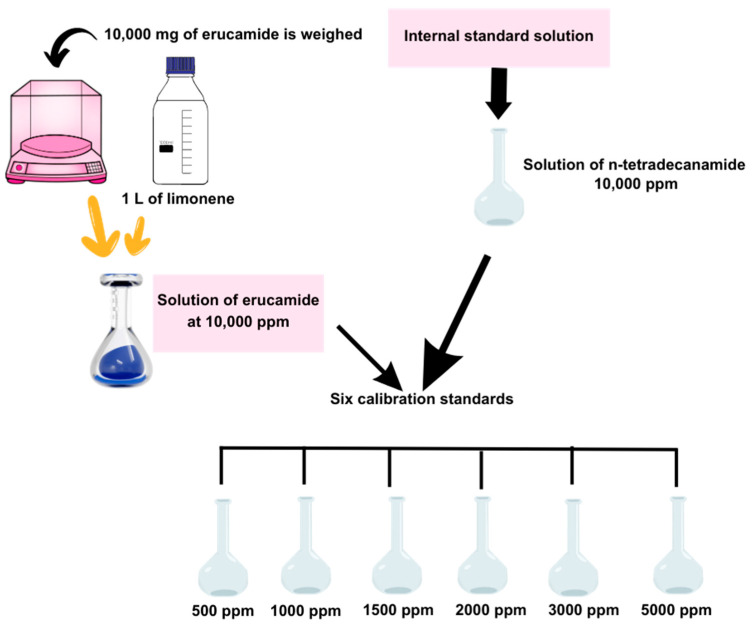
Calibration curve preparation.

A stock solution of erucamide at 10,000 ppm was prepared (10,000 mg of erucamide is weighed and 1 L of limonene was added). In another vessel, an internal standard solution of n-tetradecanamide at 10,000 ppm was prepared. Using the erucamide stock solution and the internal standard solution, six calibration standards were developed with concentrations of 5000, 3000, 2000, 1500, 1000, and 500 ppm erucamide (see Figure 2).

### 2.3. Preparation of PP Samples with Different Concentrations of Erucamide

**Figure 3:**
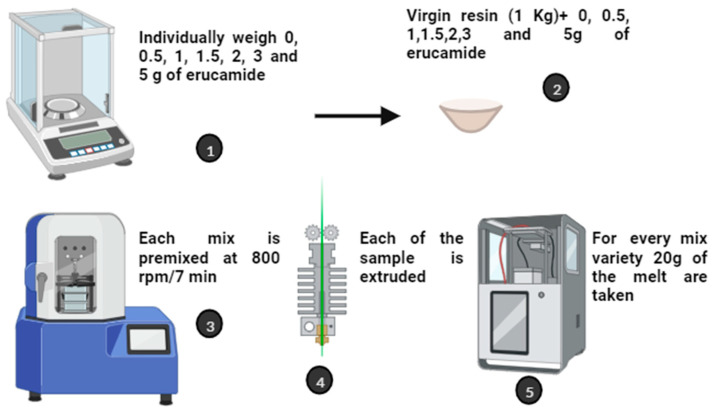
Preparation of PP samples with different concentrations of erucamide.

The PP and erucamide samples were prepared as follows: (1) Individually, 0.0, 0.5, 1, 1.5, 1.5, 2, 3, and 5 g of erucamide were weighed. (2) Each amount of erucamide was mixed with 1 kg of virgin PP resin. (3) Each mixture was premixed at 800 rpm x 7 min and using a standard Prodex Henschel 115JSS mixer (Federal Equipment Company, NJ, EE.UU). Each sample was then extruded in a Welex-200 24 extruder (KD Capital Equipment, LLC, CA, USA). The extruder operated with five temperature zones along the entire extrusion path. The temperatures were 190, 195, 200, 210, 210, and 220 °C. In this way, uniform mixing was achieved. At the outlet of the extruder, a PP-Erucamide melt mix was produced. For each melt type, 20 g of melt was fed to a CARVER 3895 hot press (SPECTRA SERVICES, INC., NY 14519, EE.UU). In this CARVER machine, the samples were compressed to form films of 300 mm diameter and ≈100 µm thickness. The films obtained in the experiment were identified as PP (0 ppm erucamide), PP2 (500 ppm erucamide), PP3 (1000 ppm erucamide), PP4 (1500 ppm erucamide), PP5 (2000 ppm erucamide), PP6 (3000 ppm erucamide), and PP7 (5000 ppm erucamide) (Figure 3).

### 2.4. Extraction of Erucamide from PP Samples

**Figure 4:**
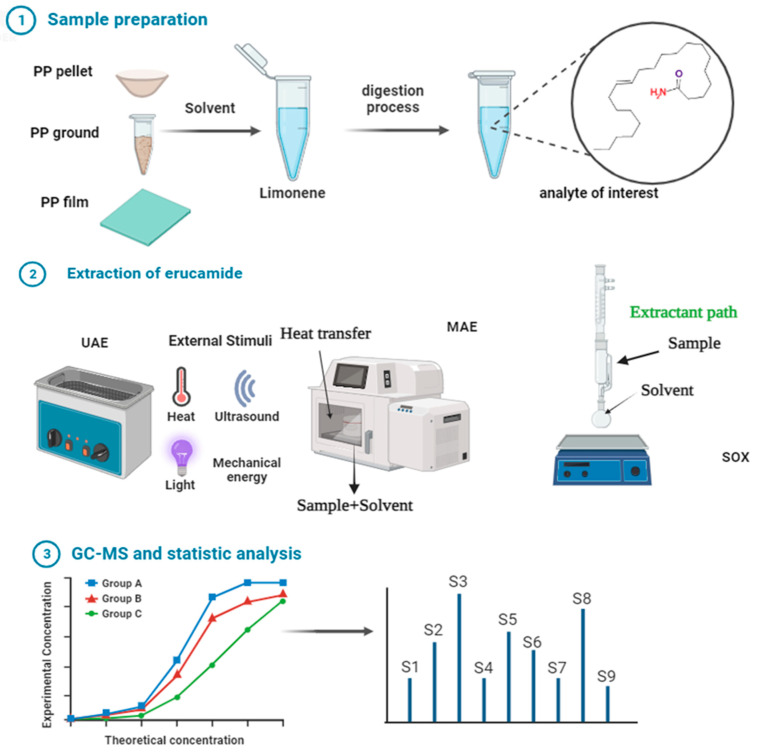
Erucamide extraction by microwave, Soxhlet, and ultrasound and quantification by GC-MS.

We obtained PP samples in the form of pellets, films, and grinds, to which erucamide was added. Three different extraction solvents were used, the first one being cyclohexane. The second option (which proved to be more efficient) was dichloromethane. The third option was limonene. Using each of the solvents, 3 different extraction methods were tested: Soxhlet, ultrasound (conventional laboratory sonic bath), and microwave oven (high-power, programmable laboratory microwave oven). Figure 4 shows the outline of the methodology followed in the investigation.

For the ultrasonic bath, 3 g of PP was added to a 30 mL vial, and then 20.0 mL of the internal standard solution was added using a 5.0 mL micropipette. For each assay, 5 replicates were performed. For three hours, the sonication procedure was carried out in an ultrasonic bath. The temperature was kept under control during the sonication process up to a maximum of 50 °C. After the sonication process was finished, the vials were taken out of the ultrasonic bath and left outside for a duration of 10 min. Disposable PTFE syringe filters were used to filter the extracted erucamide sample solutions. Crushed and pelleted PP and films were extracted for 90 and 60 min in the ultrasonic bath, with the solution agitated for at least 30 s every 10 min.

For the microwave oven, five grams of PP resin was extracted using cyclohexane, dichloromethane, and limonene. It was found that only heating the solution in the microwave oven for 25 min at 50% power was required to extract the slip agent, this process was also performed by heating for 45 min at 25% power with stirring every 5 min. Six different extractions were performed with the resin, pellets, and ground PP, and the average results for ultrasonic, Soxhlet, and microwave extraction are shown in Table 1. The microwave oven provided a very rapid means of extracting the erucamide from the crushed resin. The ultrasonic bath provided an economical and relatively fast way to extract the additives. The Soxhlet method of extraction with these polypropylene resins took at least 7 h to extract most of the additives. In our case, Soxhlet extraction was carried out for 1440 and 720 min and possibly took more than 24 h to fully recover the additive.

### 2.5. Statistical Analysis

For the present study, a statistical evaluation of the recovery of erucamide in polypropylene samples was conducted. For this purpose, the Minitab software, recognized for its capability of advanced statistical analysis, was used. Given the multifactorial nature of the study, which involved various variables such as extraction techniques (microwave, Soxhlet, and ultrasound), solvents (dichloromethane, cyclohexane, and limonene), and types of polypropylene (ground, pellets, films), a multivariable graphical analysis was performed. This approach allowed for the exploration of interactions and complex relationships among the variables involved in the recovery of erucamide.

#### Multivariable Graphical Analysis

The multivariable graphical analysis was carried out to visualize and understand the relationship between different extraction techniques, solvents, and forms of polypropylene used in the study. Graphical representation techniques were employed to examine the dependency among variables and explore possible patterns and trends.

## 3. Results

### 3.1. Quantification and Recovery of Erucamide by GC-MS

In this study, the presence of erucamide in samples of polypropylene (PP) was determined using a method called the internal standard method. The validity of the GC-MS method was thoroughly tested and confirmed. Both the standard solutions and the samples were analyzed twice to ensure accuracy. The calibration curve, which plots the concentration of erucamide against the instrument response, demonstrated a straight-line relationship within the specified range. The coefficient of determination, a statistical measure of how well the data fit the curve, exceeded 0.999, indicating a highly reliable correlation.

In order to conduct the analysis, we created erucamide solutions with six different concentrations (500, 1000, 1500, 2000, 3000, and 5000 parts per million). These solutions were prepared using an internal standard. The erucamide extracts, obtained from various PP samples such as PP film, PP pellets, and ground PP, were then subjected to analysis using the GC-MS method, following the procedure outlined in Section 2.2.

In the study carried out to evaluate the recovery of erucamide, different extraction techniques (microwave, Soxhlet, and ultrasound), three different solvents (cyclohexane, dichloromethane, and limonene), different forms of polypropylene (films, ground, and pellets) and different extraction times were used. To analyze the results, a variability graph was created to identify the differences in the means and variations in the recovery of erucamide at the combined levels.

**Figure 5:**
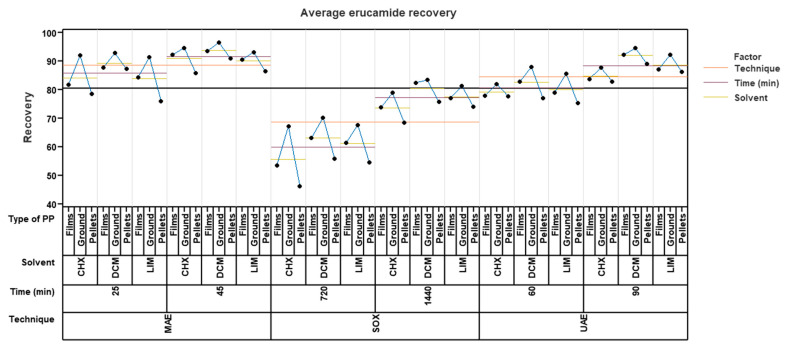
Average recovery of erucamide for microwave (MAE), Soxhlet (SOX), and ultrasound (UAE) with cyclohexane (CHX), dichloromethane (DCM), and limonene (LIM).

Figure 5 shows the relationship between the percentage of erucamide recovery and the variables mentioned above. When analyzing the graph, it was observed that the microwave extraction technique, with a time of 25 min and using ground polypropylene, achieved the highest recovery percentages. However, no significant differences were observed with respect to the solvent used, since the recovery percentages were close to each other. Specifically, dichloromethane obtained a recovery of 92.68%, cyclohexane obtained a recovery of 91.87%, and limonene obtained a recovery of 91.37%. In the case of using an extraction time of 45 min, better results were obtained using ground polypropylene with dichloromethane as a solvent, achieving a recovery percentage of 96.36%. Cyclohexane was in second place with a percentage of 94.57%, and limonene was in last place with 93.05%.

Using ultrasound, better results were obtained in an extraction time of 90 min and likewise in ground pp using dichloromethane as a solvent with a recovery percentage of 94.38%, followed by limonene in ground pp with a percentage of 92.17% and finally cyclohexane in ground pp with a percentage of 87.59%. It can be observed that limonene obtained better results than cyclohexane regardless of the presentation of the polymer. In an extraction time of 60 min, the solvent with the best recovery percentage was dichloromethane in ground pp with 87.88%, followed by limonene with 85.56% and, finally, Cyclohexane with 81.97%.

Finally, Soxhlet extraction obtained lower recovery results compared to microwave and ultrasound. The highest percentage reached using this technique was with a time of 1440 min, using dichloromethane as a solvent and ground pp, with 83.29% recovery. This was followed by limonene in ground pp with 81.18% and lastly by cyclohexane with 78.77%.

This may be because the microwave extraction technique uses microwaves to selectively heat the solvent and sample. This allows for faster and more efficient heat transfer, which speeds up the extraction process. In contrast, the ultrasound and Soxhlet techniques may require more time to reach the right temperature and achieve a complete extraction, as demonstrated in the experimental design, since the Soxhlet extraction needed a time of 1440 min to achieve good recoveries that were well below the recovery percentages obtained by microwave, which only took 45 min. Another reason is that the microwave extraction technique can provide greater agitation and turbulence in the sample, which improves the interaction between the solvent and the analyte. This facilitates erucamide extraction and improves recovery efficiency. And as is known, the microwave extraction technique was able to achieve comparable or better results in a shorter extraction time compared to the ultrasonic and Soxhlet techniques. A shorter extraction time can minimize the degradation or loss of the analyte during the process and improve the recovery, as detailed in Section 2.3, and also allows greater control of the extraction conditions, such as temperature and pressure. This allows the conditions to be optimized to maximize the recovery of the erucamide and minimize any possible interference or degradation of the analyte. Although dichloromethane showed slightly higher recovery percentages, the difference was not significant enough to completely rule out limonene as a solvent option.

In these cases, it is important to weigh the additional benefits of limonene as a green solvent, such as lower toxicity and reduced environmental impact. Furthermore, the choice of solvent depends on other factors, such as current environmental regulations, specific application requirements, and personal or company preferences. If sustainability is valued and the minimization of environmental impact is sought, the choice of limonene as a green solvent may be better aligned with these objectives. In addition to not showing a significant difference compared to dichloromethane, limonene also outperformed cyclohexane in terms of percent erucamide recovery. This is another important consideration when choosing limonene as a solvent.

Cyclohexane, being a toxic solvent, may pose occupational health and safety concerns. In addition, this solvent had a lower contribution to the extraction of erucamide in polypropylene, unlike dichloromethane and limonene. Opting for limonene as a safer and less toxic alternative may be beneficial for both operators and the environment. When selecting solvents, it is essential to consider both extraction efficiency and aspects related to safety and environmental impact. In this case, limonene not only demonstrated erucamide recovery comparable to dichloromethane, but also can avoid the risks associated with the use of cyclohexane.

Nielson performed the extraction and quantification of a series of polyolefin additives including erucamide in low-density polyethylene (LDPE) using a 98:2 methylene chloride/isopropanol mixture as extraction solvent, using ground resins (20 mesh, under liquid nitrogen), for 20 min. The highest percentage of recovery obtained by the author was 91% using a microwave oven. For ultrasonic extraction, he used a 75:25 mixture of methylene chloride/cyclohexane, with which he obtained a 94% recovery of erucamide. The author concluded that the erucamide recoveries are similar and very satisfactory for both extraction techniques.

We can say that both the aforementioned study and the present study achieved quite high recovery percentages using different techniques and solvents. In the Nielson study, recoveries of around 91% were obtained using microwave extraction, and recoveries of around 94% were obtained using ultrasound extraction. In this research, recovery percentages higher than 90% were obtained in most of the conditions evaluated, for microwave, ultrasound, and Soxhlet, using different solvents, extraction times, and polymer forms. It is important to note that the experimental conditions such as the type of polymer used, particle size, solvent mixtures, and extraction times vary between studies. These variations clearly influence the results obtained and make a direct comparison between studies difficult.

### 3.2. Identification of Erucamide by Mass Spectrometry

The erucamide extraction was performed with the objective of obtaining as much of the original substance as possible without significant contamination. However, if the erucamide has been degraded during the process, the recovery percentages will be lower. The extracted erucamide was analyzed by GC-MS to follow up the original erucamide and observe its transformation into degraded byproducts that may be more difficult to recover or detect during the analysis. The GC-MS analysis mentioned above helps to identify the degraded byproducts and determine whether the erucamide has undergone significant degradation. The data analysis was conducted with the understanding that the compounds being examined are degradation products of erucamide.

To identify and measure these compounds, the fragmentation spectrum of erucamide was used as a reference. This spectrum provided valuable information that aided in the identification and quantification of the degradation products. In this way, it was possible to relate the peaks and features observed in the spectra of the analyzed compounds with the structure and fragments present in the erucamide.

Erucamide is susceptible to oxidative degradation due to its chemical structure and the presence of functional groups. During the extraction process, especially when solvents such as cyclohexane, dichloromethane, and limonene are used, conditions that favor the oxidation of erucamide can occur. The oxidation of erucamide can lead to the formation of degradation products, which could affect the solubility and extraction efficiency of erucamide in the solvents used. These degradation products could have a lower ability to interact with the solvents, resulting in a lower erucamide extraction yield.

In addition, oxidative degradation of erucamide may lead to the formation of compounds with different properties, such as the generation of more polar compounds. These modified compounds could have a lower affinity for the solvents used in the extraction, which would make their separation from the polypropylene resin more difficult and, consequently, could reduce the extraction yield. It is important to note that the oxidative degradation of erucamide can be influenced by several factors, such as temperature, the presence of catalysts, the duration of the extraction process, and the storage conditions of the polypropylene resin. A higher degree of oxidative degradation of erucamide may be indicative of a less efficient extraction process and therefore a lower yield.

To optimize extraction yield, it is important to consider measures to minimize oxidative degradation of erucamide during the extraction process and storage of polypropylene resin. The results of this study indicated that measuring the degree of oxidative degradation of erucamide in the polyolefin resin is indirectly a measure of the erucamide extraction performance imparted to the polyolefin resin. Mass spectrometry techniques were employed to verify the proposed mechanisms responsible for the generation of degradation species. Through the utilization of these techniques, the proposed mechanisms were successfully validated.

**Figure 6:**
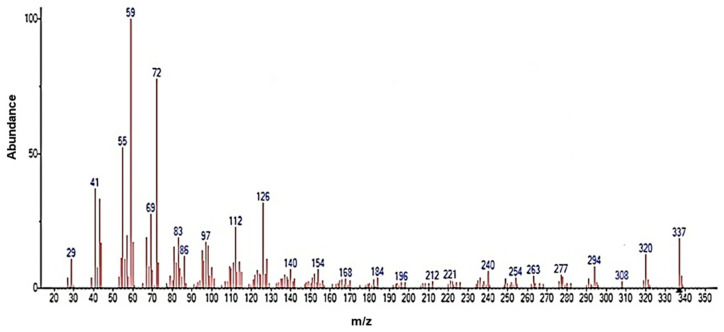
MS spectrum of the recovered erucamide.

**Figure 7:**
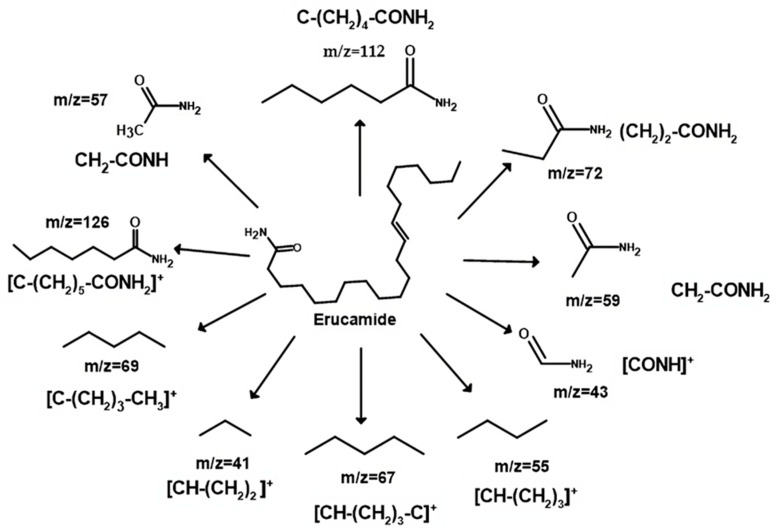
Recovered erucamide fragmentation mechanism.

Figure 6 shows the mass spectrum obtained, which revealed a characteristic pattern of linear hydrocarbons. To better understand the structure of erucamide and the fragmentation patterns observed, a fragmentation mechanism is recommended in Figure 7. The loss of small molecules, such as hydrogen, results in a decrease in the total amount of erucamide extracted because the more fragmentation occurs and the more molecules are lost, the lower the final amount of erucamide obtained in the extraction process. Table 2 shows the byproducts of erucamide degradation.

Upon fragmentation, the erucamide leads to the breaking of important bonds in its structure, such as the H-H bond, as observed in the fragmented ions with m/z 41, 55, 59, 72, 112, and 126, which correspond to the fragmentation of the chain near the amide group and the breaking of hydrogen bonds. These bonds are an integral part of the molecule and contain valuable information about its composition and properties. When they are broken, this information is lost and the precise identification of the erucamide and the interpretation of its structure become difficult.

#### 3.2.1. Determination of Thermo-Oxidative Degradation Byproducts of Erucamide

**Figure 8:**
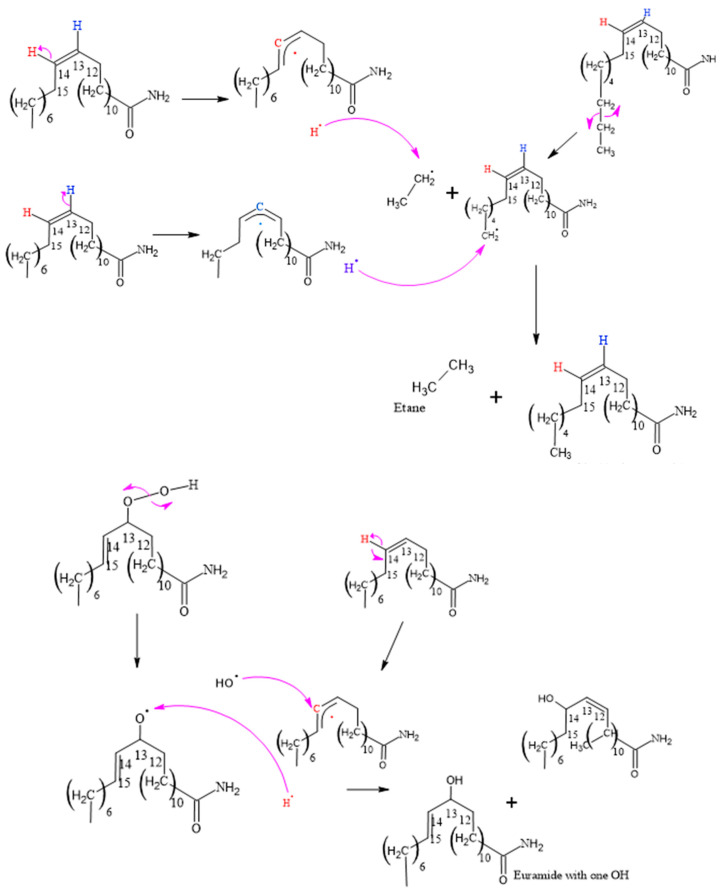
Formation of Cis 11-eicosenamide and erucamide with one OH.

**Figure 9:**
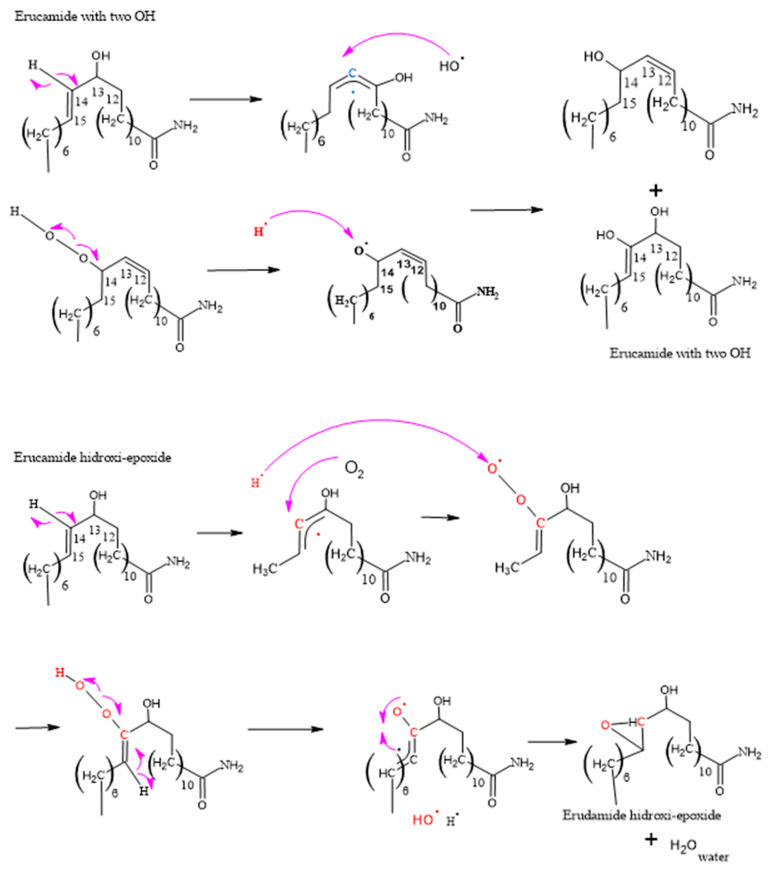
Formation of the degradation of erucamide with two OH groups and erucamide hydroxy-epoxide.

**Figure 10:**
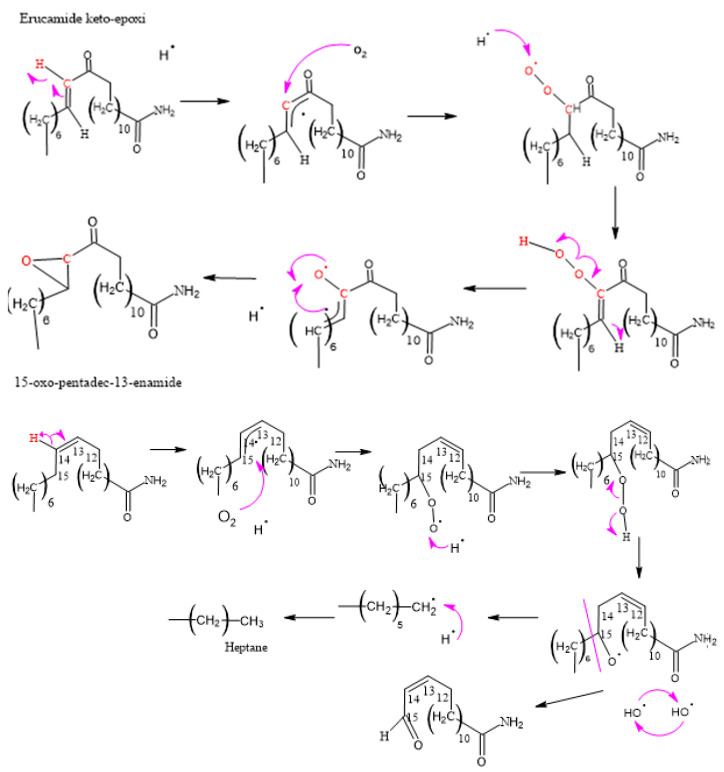
Formation of erucamide keto-epoxide and 15-oxo-pentadec-13-enamide.

**Figure 11:**
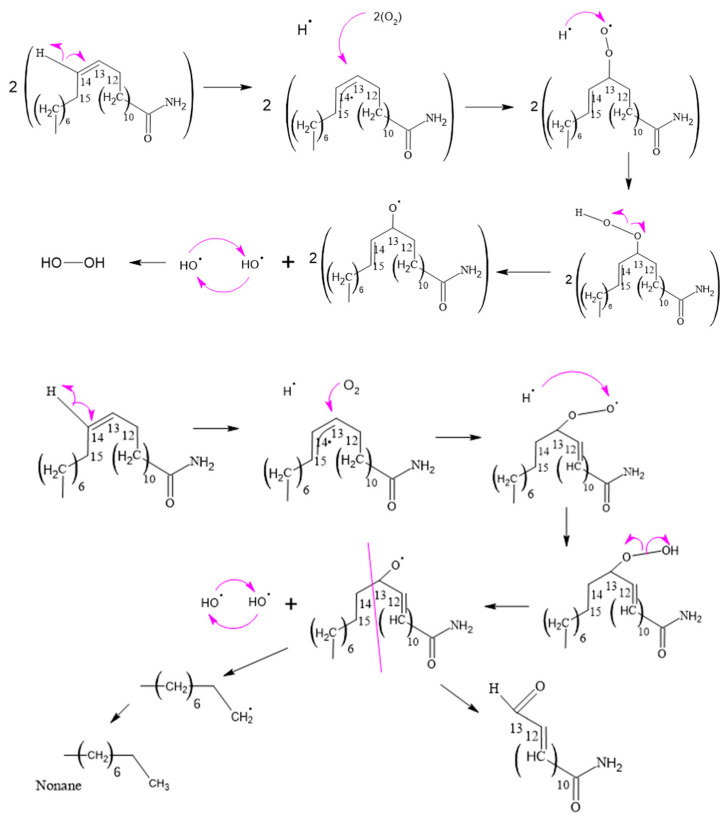
Formation of hydrogen peroxide and 13-oxo-pentadec-11-enamide.

**Figure 12:**
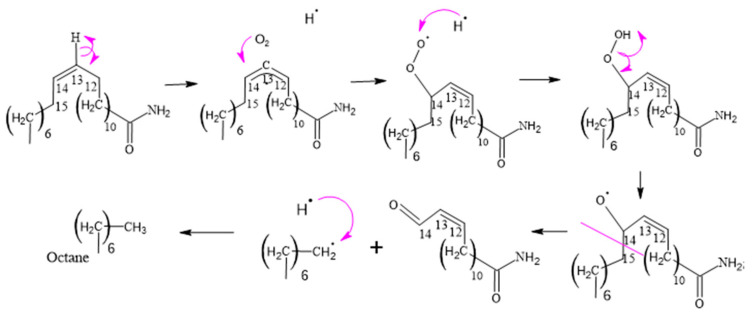
Formation of 14-oxotetradec-12-enamide.

Figure 8, Figure 9, Figure 10, Figure 11 and Figure 12, which illustrate the mechanisms underlying the formation of erucamide degradation products, are presented below. These mechanisms are characterized by the abundant presence of hydrogen and hydroxyl radicals, the simultaneous occurrence of the generation of these radicals in several different molecules and at different times, and the random nature of the reactions that occur between these radicals and the macro radicals of erucamide. It is important to note that many of these mechanisms present advanced starting species or radicals already formed because the process through which they reach that state, as described in Figure 8, is common to all of them. However, it is necessary to distinguish between the degradation routes that originate in the first part of Figure 8, where the macroradical erucamide and the hydrogen radical are generated; the second part of the route in Figure 8, where, after the union of oxygen and a hydrogen radical to form the peroxide function, the scission of the peroxo (O-O) bond occurs, thus generating the hydroxyl radical and an oxygen radical attached to the carbon chain of the erucamide; and the third part of the mechanism in Figure 8, where the carbon chain of the erucamide is broken, generating a formyl group and an alkenyl (α) free radical or an aldehyde and a radical with the amido (β) group characteristic of erucamide.

#### 3.2.2. Formation of Erucamide with Two OH Groups and Hydroxy-Epoxide

Figure 9 shows one of the analyses performed on two of the degradation products of erucamide, which allowed us to obtain significant results in terms of understanding the chemical reactions involved. The presence of hydrogen and hydroxyl radicals in abundance suggests that they play a fundamental role in the formation of the degradation products. Moreover, the simultaneity in the generation of these radicals in different molecules and at different times provides an enabling scenario for a series of chain reactions and cascade reactions.

The randomness of the interactions between the hydrogen, hydroxyl, and macro radicals of erucamide introduces a complexity factor into the system, leading to the formation of a variety of degradation products, such as erucamide hydroxy-epoxide. The diversity of the products obtained can be attributed to the different positions at which the radicals bind to the carbon chain of the erucamide, resulting in structural modifications and the formation of new chemical bonds.

#### 3.2.3. Formation of Erucamide Keto-Epoxide and the 15-Oxo-pentadec-13-enamide

It is important to note that, due to the complexity of the mechanisms and chemical reactions involved, the formation of erucamide degradation products does not follow a specific and predictable pattern. Instead, the random nature of the reactions and the interaction between radicals contribute to the diversity of the final products. Therefore, a thorough and detailed experimental approach is required to fully understand the degradation products generated under specific conditions. The compounds shown in Figure 7 display additional product ions where the loss of 18 units (-H_2_O) occurs. Additionally, they exhibit characteristic fragments that arise from the presence of amide bonds. Typically, the loss of 18 units is not highly specific because it is commonly observed in compounds that possess functional groups containing oxygen. However, this loss of water molecules is frequent in aliphatic alcohols with a relatively high ratio [55]. Hence, these compounds are generated as a result of the oxidation and degradation process of erucamide. They have a relatively short aliphatic chain and consist of an amide group along with one or more hydroxyl groups.

#### 3.2.4. Hydrogen Peroxide and 13-Oxo-pentadec-11-enamide Formation

The process of lipid oxidation is intricate, and unsaturated fatty acids are especially prone to oxidative degradation. The creation of various degradation products is influenced by multiple factors, including the presence of oxygen, ultraviolet and visible light exposure, heat, and metal catalysts. These factors play a role in the generation of undesirable oxidation products.

In the course of this process, hydrogen peroxides are formed by the extraction of hydrogen from peroxy radicals, as illustrated in Figure 11. Unsaturated fatty acids, with their double bonds, are more susceptible to hydrogen abstraction due to their lower dissociation energy in comparison to saturated aliphatic chains. The instability of hydroperoxides causes them to decompose into alkoxy radicals, which undergo β-scission on both sides of the alkoxy carbon, leading to the formation of aldehydes, ketones, carboxylic acids, alcohols, epoxides, and hydrocarbons.

#### 3.2.5. Formation of 14-Oxotetradec-12-enamide and 15-Amino-15-oxopentadec-2-enoic Acid

When the C14 of erucamide undergoes oxidation, several major products are generated. These include nonanal, 14-oxotetradec-12-enamide, and 13-oxotridecanamide. In addition, 13-oxotridecanamide is converted to 13-amino-13-oxotridecanoic acid, while 14-oxotetradec-12-enamide is converted to 14-amino-14-oxo-tetradec-2-enoic acid.

In Figure 12, an erucamide derivative is shown that features a previously formed formyl group. In this process, the formyl group undergoes a cleavage of the carbon–hydrogen bond within the same group, resulting in the formation of a radical. This radical is in a reactive position and is attacked by a hydroxyl radical from another molecule containing a peroxo bond.

This reaction allows the stabilization of both radicals since a new chemical connection is formed between them, thus generating an acid group. In other words, the formyl group undergoes a cleavage in its carbon–hydrogen bond, which gives rise to a radical that combines with a hydroxyl radical to form a new acid group in the molecule.

#### 3.2.6. Formation of 13-Amino-13-oxotridecanoic Acid and 15-Amino-15-oxopentadeca-enoic Acid

**Figure 13:**
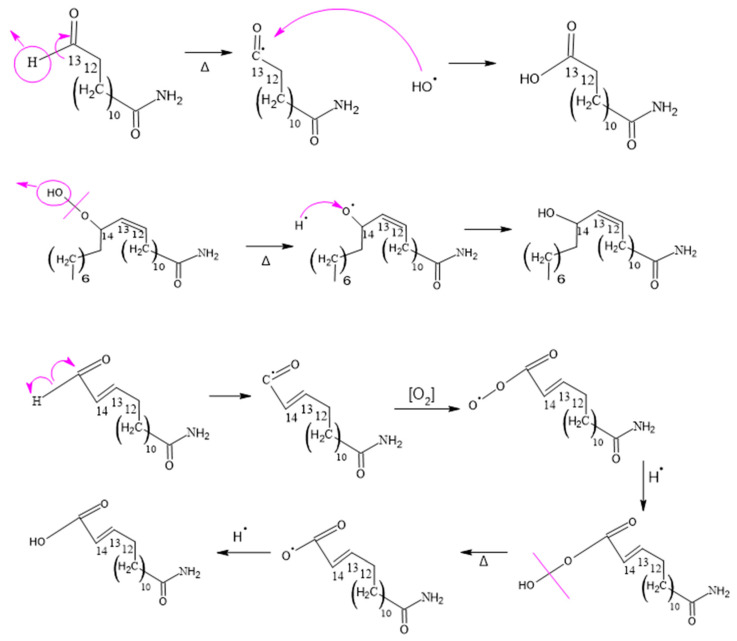
Formation of 13-amino-13-oxotridecanoic and 15-Amino-15-oxopentadeca-enoic Acid.

The mechanism illustrated for these compounds observed in Figure 13 is similar to that observed for 15-amino-15-oxo-pentadec-2-enoic acid. However, in this case, it is a chain with 13 and 14 carbons, which is a variant of the two possible carbons where oxygen can attack during the first carbon–hydrogen scission in the double bond of the original erucamide molecule.

**Figure 14:**
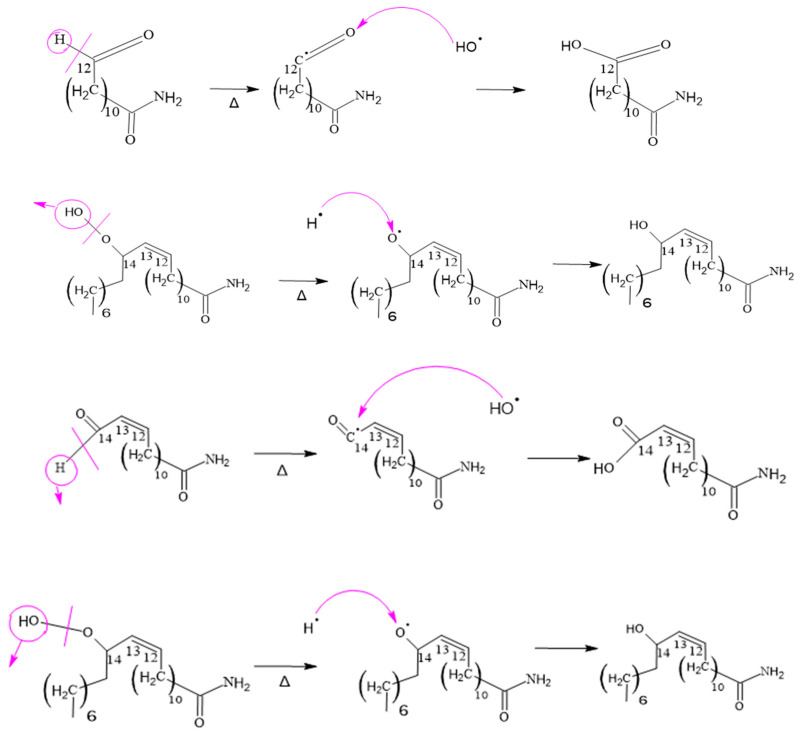
Formation of 12-Amino-12-oxododecaenoic Acid and 14-Amino-14-oxo-tetradec-2-enoic Acid.

This mechanism also applies to other similar compounds, such as 12-amino-12-oxododecanoic acid, 14-amino-14-oxotetradecanoic acid and 14-amino-14-oxotetradec-2-enoic acid. In these cases, the difference is in the length of the carbon chain, but the process of carbon–hydrogen scission and formation of amino groups and acids is analogous to that described above. As shown in Figure 14.

### 3.3. Percentage Analysis of Erucamide Degradation Byproducts

**Figure 15:**
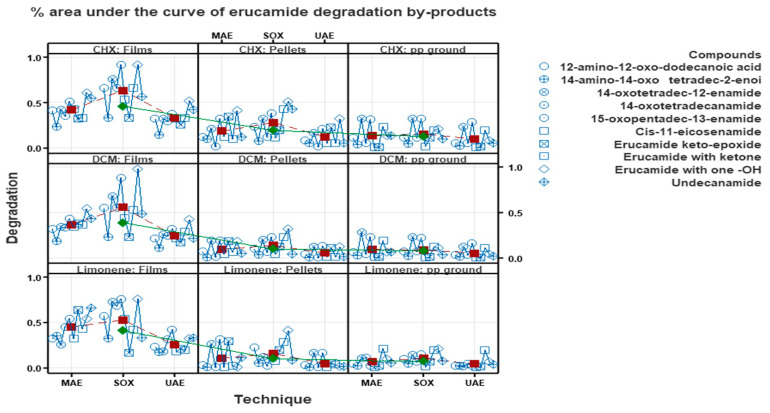
Percent area under the curve of erucamide degradation byproducts without the concentration of erucamide.

Figure 15 illustrates the variability of different methods, solvents and forms of polypropylene related to erucamide concentrations. The differences in erucamide byproduct concentrations between the Soxhlet and microwave techniques, as well as between the solvents used, can be attributed to the different extraction conditions and the specific properties of the solvents in terms of their ability to extract and retain the degradation byproducts.

In the case of the Soxhlet technique, which involves prolonged and continuous extraction, a higher concentration of erucamide byproducts may have been obtained due to the longer duration and greater contact with the solvent. In addition, cyclohexane and dichloromethane, used as solvents in the Soxhlet technique, may have a greater ability to extract erucamide degradation byproducts compared to limonene.

The difference in the ability of the solvents (cyclohexane, dichloromethane and limonene) to extract erucamide degradation byproducts can be attributed to their chemical and physical properties. Cyclohexane and dichloromethane are organic solvents of medium to low polarity. These solvents are known for their ability to dissolve organic compounds and can efficiently extract erucamide degradation byproducts. This is because these solvents are lipid soluble and have a higher affinity for organic compounds. On the other hand, limonene is a naturally occurring solvent with relatively high polarity. Although limonene can also dissolve organic compounds, its ability to extract erucamide degradation byproducts may be lower compared to cyclohexane and dichloromethane. This is because limonene has a lower solubility in lipids and may have a lower affinity for organic compounds compared to the other solvents mentioned.

On the other hand, the microwave technique generally involves a faster and more efficient extraction due to the use of microwave radiation to heat the sample. Although this technique provided higher erucamide recovery percentages compared to the ultrasound technique, it is possible that the shorter extraction times used in the microwave technique did not allow a complete extraction of the degradation byproducts.

The difference in erucamide degradation rates between polypropylene (PP) in film, milled and pellet form may be related to the accessibility and physical structure of the material. When PP is in film form, it has a larger exposed surface area compared to ground PP and pellets. This means that more surface area is available for solvents and degradation conditions to interact with the erucamide and its byproducts. As a result, it is possible that more degradation of erucamide may occur in the PP in film form, which would be reflected in higher degradation percentages. On the other hand, ground PP and pellets have a more compact physical structure compared to film. This may hinder the access of solvents and degradation conditions to the erucamide and limit the interaction between them. As a result, less degradation of erucamide may occur in the milled PP and pellets, which would be reflected in lower degradation percentages, as shown in Figure 15.

## 4. Conclusions

The results of the study show that the use of ground polypropylene instead of polypropylene forms in films and pellets can improve erucamide recovery due to higher contact surface area, higher permeability, smaller particle size and higher homogeneity of the material. In addition, the microwave extraction technique was found to be more effective than ultrasound and Soxhlet techniques, as it allowed shorter extraction times and higher recovery efficiency. In microwaves with a time of 25 min and using ground polypropylene, the highest erucamide recovery was achieved, with percentages higher than 96.4%. With a time of 45 min and using ground polypropylene, the recovery reached 96.36%. Using the ultrasound technique, with an extraction time of 90 min and using ground polypropylene, a recovery of 94.38% was obtained. Using the Soxhlet extraction technique, with an extraction time of 1440 min and using ground polypropylene, a recovery of 83.29% was achieved. These results indicate that the microwave extraction technique in combination with ground polypropylene obtained the highest recovery percentages in relatively short extraction times. Although dichloromethane showed a slight advantage in terms of recovery, the use of limonene as a solvent was also viable and offered additional benefits, such as lower toxicity and reduced environmental impact. In contrast, cyclohexane raises occupational health and safety concerns due to its toxicity. Therefore, selecting limonene as a safer and environmentally friendly alternative may be beneficial. In general, it is important to consider both extraction efficiency and safety and environmental impact issues when choosing the appropriate solvent.

## Figures and Tables

**Table 1:** Erucamide recovery percentage according to the experimental design.

Technique	Time (min)	Temperature (°C)	Solvent	Form of PP	Erucamide Recovery %					
					PP2	PP3	PP4	PP5	PP6	PP7
Microwave	45	117	Cyclohexane	Pellets	85.40	82.82	85.23	87.25	86.85	86.84
Microwave	45	117	Cyclohexane	Films	87.5	92.65	94.67	92.25	93.17	92.45
Microwave	45	117	Cyclohexane	PP-ground	91.78	94.50	96.67	95.25	95.33	93.88
Microwave	25	117	Cyclohexane	Pellets	76.50	76.35	72.30	78.83	83.68	83.54
Microwave	25	117	Cyclohexane	Films	77.40	81.70	79.67	85.95	81.50	84.20
Microwave	25	117	Cyclohexane	PP-ground	87.39	92.30	93.17	93.68	91.93	92.76
Soxhlet	1440	90	Cyclohexane	Pellets	65.80	68.95	67.83	65.95	70.49	71.54
Soxhlet	1440	90	Cyclohexane	Films	74.10	70.85	72.00	72.69	73.91	78.59
Soxhlet	1440	90	Cyclohexane	PP-ground	79.00	78.33	78.88	78.20	79.5	78.73
Soxhlet	720	90	Cyclohexane	Pellets	45.50	39.45	38.70	48.45	53.78	51.42
Soxhlet	720	90	Cyclohexane	Films	52.15	56.95	49.57	56.88	53.78	51.42
Soxhlet	720	90	Cyclohexane	PP-ground	65.80	66.40	68.83	65.20	67.11	69.55
Ultrasound	90	50	Cyclohexane	Pellets	83.80	81.14	80.8	84.69	82.07	84.17
Ultrasound	90	50	Cyclohexane	Films	86.00	82.92	80.05	83.45	79.83	89.61
Ultrasound	90	50	Cyclohexane	PP-ground	88.56	88.52	82.84	87.62	85.55	92.46
Ultrasound	60	50	Cyclohexane	Pellets	77.50	77.37	75.23	79.47	77.28	78.7
Ultrasound	60	50	Cyclohexane	Films	80.40	78.26	74.86	78.85	76.16	78.3
Ultrasound	60	50	Cyclohexane	PP-ground	84.28	81.00	80.25	82.36	82.54	81.4
Microwave	45	117	Dichloromethane	Pellets	89.14	90.26	92.39	91.44	91.03	90.67
Microwave	45	117	Dichloromethane	Films	94.92	94.28	92.79	93.68	91.65	93.4
Microwave	45	117	Dichloromethane	PP-ground	96.44	96.54	97.29	94.94	94.74	98.35
Microwave	25	117	Dichloromethane	Pellets	86.07	87.38	87.69	86.84	86.76	88.51
Microwave	25	117	Dichloromethane	Films	89.90	84.75	86.37	87.97	87.08	89.88
Microwave	25	117	Dichloromethane	PP-ground	92.60	90.52	93.48	92.37	93.19	93.92
Soxhlet	1440	90	Dichloromethane	Pellets	78.60	72.60	74.53	79.18	71.98	76.93
Soxhlet	1440	90	Dichloromethane	Films	78.60	77.85	87.33	84.20	81.98	84.21
Soxhlet	1440	90	Dichloromethane	PP-ground	82.60	82.15	84.10	82.50	85.17	83.23
Soxhlet	720	90	Dichloromethane	Pellets	53.50	59.40	52.6	60.05	56.17	53.13
Soxhlet	720	90	Dichloromethane	Films	60.60	62.95	65.23	60.53	66.63	62.52
Soxhlet	720	90	Dichloromethane	PP-ground	68.60	69.50	72.67	68.15	69.38	72.80
Ultrasound	90	50	Dichloromethane	Pellets	87.88	89.42	88.04	89.62	88.85	89.41
Ultrasound	90	50	Dichloromethane	Films	92.84	91.76	93.25	91.81	88.47	95.10
Ultrasound	90	50	Dichloromethane	PP-ground	94.68	93.98	95.51	93.68	91.45	96.99
Ultrasound	60	50	Dichloromethane	Pellets	74.10	78.75	77.87	79.25	78.58	72.9
Ultrasound	60	50	Dichloromethane	Films	82.40	84.30	80.37	84.75	79.63	84.51
Ultrasound	60	50	Dichloromethane	PP-ground	87.90	85.50	85.77	91.49	88.53	88.10
Microwave	45	117	Limonene	Pellets	86.07	87.23	84.87	86.08	86.47	87.21
Microwave	45	117	Limonene	Films	90.20	90.80	89.90	90.63	90.00	91.10
Microwave	45	117	Limonene	PP-ground	94.10	89.40	94.27	92.50	94.18	93.85
Microwave	25	117	Limonene	Pellets	74.70	78.80	71.57	78.85	76.50	75.25
Microwave	25	117	Limonene	Films	81.40	82.80	84.33	87.18	85.27	84.10
Microwave	25	117	Limonene	PP-ground	91.55	89.25	91.87	90.88	91.84	92.84
Soxhlet	1440	90	Limonene	Pellets	76.40	72.10	72.53	76.64	71.26	74.43
Soxhlet	1440	90	Limonene	Films	77.80	76.83	78.67	76.08	73.20	79.20
Soxhlet	1440	90	Limonene	PP-ground	79.8	79.93	82.02	80.63	82.57	82.14
Soxhlet	720	90	Limonene	Pellets	52.25	58.45	50.80	59.40	53.78	51.71
Soxhlet	720	90	Limonene	Films	58.80	61.38	62.98	59.64	64.32	61.27
Soxhlet	720	90	Limonene	PP-ground	64.20	67.45	70.50	67.10	68.23	68.09
Ultrasound	90	50	Limonene	Pellets	84.76	83.00	83.65	88.34	88.55	88.20
Ultrasound	90	50	Limonene	Films	83.28	86.7	87.25	85.80	86.63	92.58
Ultrasound	90	50	Limonene	PP-ground	91.08	91.78	91.39	92.33	89.99	96.46
Ultrasound	60	50	Limonene	Pellets	74.10	79.40	74.03	72.03	75.5	76.61
Ultrasound	60	50	Limonene	Films	78.10	77.95	79.67	76.55	80.72	79.74
Ultrasound	60	50	Limonene	PP-ground	83.20	89.05	88.30	84.65	80.27	87.91

**Table 2:** Degradation profile of erucamide in different solvents.

					% of Degraded Fragments/Type of Solvent/Extraction Technique					
Tr (Min)	Compound	Mass	Formula	Fragments (m/z)	Microwave		Soxhlet		Ultrasound	
3.06	12-amino-12-oxo-dodecanoic acid	228.16	C_12_H_23_NO_3_	211.13, 210.15, 184.17, 167.14	DCM	0.15	DCM	0.24	DCM	0.093
					CHX	0.2133	CHX	0.333	CHX	0.15
					LIM	0.128	LIM	0.293	LIM	0.091
4.34	14-amino-14-oxotetradecanoic acid	256.2	C_14_H_27_NO_3_	239.16, 212.2, 238.18 195.17	DCM	0.0693	DCM	0.093	DCM	0.0413
					CHX	0.1173	CHX	0.1466	CHX	0.0703
					LIM	0.124	LIM	0.136	LIM	0.063
6.58	14-oxotetradecanamide	242.2	C_14_H_27_NO_2_	225.18, 207.17, 197.2	DCM	0.126	DCM	0.226	DCM	0.0913
					CHX	0.1363	CHX	0.296	CHX	0.1156
					LIM	0.188	LIM	0.253	LIM	0.118
7.26	15-oxopentadec-13-enamide	254.21	C_15_H_27_NO_2_	239.2, 237.18, 219.17, 201.16	DCM	0.283	DCM	0.443	DCM	0.203
					CHX	0.38	CHX	0.54	CHX	0.286
					LIM	0.29	LIM	0.34	LIM	0.2
7.85	Undecanamide	186.18	C_11_H_23_NO	169.16, 158.15, 151.08	DCM	0.18	DCM	0.183	DCM	0.08
					CHX	0.266	CHX	0.36	CHX	0.1733
					LIM	0.271	LIM	0.16	LIM	0.124
8.8	Erucamide keto-epoxide	368.3	C_22_H_41_NO_3_	351.29, 352.32, 333.28, 315.27	DCM	0.1896	DCM	0.12	DCM	0.0953
					CHX	0.221	CHX	0.21	CHX	0.159
					LIM	0.316	LIM	0.136	LIM	0.089
10.78	Erucamide with ketone	352.3	C_22_H_41_NO_2_	335.29, 317.28, 307.29, 299.27	DCM	0.203	DCM	0.293	DCM	0.136
					CHX	0.216	CHX	0.4266	CHX	0.19
					LIM	0.22	LIM	0.296	LIM	0.136
11.3	Erucamide with one -OH	354.3	C_22_H_43_NO_2_	337.31, 336.33, 319.29, 309.31, 301.29	DCM	0.26	DCM	0.47	DCM	0.193
					CHX	0.383	CHX	0.546	CHX	0.306
					LIM	0.21	LIM	0.38	LIM	0.506
12	Cis-11-eicosenamide	310.3	C_20_H_39_NO	293.28, 275.27	DCM	0.129	DCM	0.1613	DCM	0.0736
					CHX	0.1846	CHX	0.2173	CHX	0.1246
					LIM	0.11	LIM	0.206	LIM	0.063
13	Erucamide (13-cis-Docosenamide)	338.3	C_22_H_43_NO	321.32, 303.31	DCM	93.55	DCM	80.43	DCM	91.819
					CHX	90.804	CHX	73.629	CHX	84.67
					LIM	89.935	LIM	77.343	LIM	88.431

## Data Availability

Not applicable.
